# Assessing Cyberbiosecurity Vulnerabilities and Infrastructure Resilience

**DOI:** 10.3389/fbioe.2019.00061

**Published:** 2019-03-29

**Authors:** Daniel S. Schabacker, Leslie-Anne Levy, Nate J. Evans, Jennifer M. Fowler, Ellen A. Dickey

**Affiliations:** ^1^Argonne National Laboratory (DOE), Strategic Security Sciences Division, Lemont, IL, United States; ^2^Argonne National Laboratory (DOE), Decision and Infrastructure Sciences Division, Lemont, IL, United States

**Keywords:** cyberbiosecurity, vulnerability, resilience, risk, convergence, emerging, converging, technology

## Abstract

The convergence of advances in biotechnology with laboratory automation, access to data, and computational biology has democratized biotechnology and accelerated the development of new therapeutics. However, increased access to biotechnology in the digital age has also introduced additional security concerns and ultimately, spawned the new discipline of cyberbiosecurity, which encompasses cybersecurity, cyber-physical security, and biosecurity considerations. With the emergence of this new discipline comes the need for a logical, repeatable, and shared approach for evaluating facility and system vulnerabilities to cyberbiosecurity threats. In this paper, we outline the foundation of an assessment framework for cyberbiosecurity, accounting for both security and resilience factors in the physical and cyber domains. This is a unique problem set, but despite the complexity of the cyberbiosecurity field in terms of operations and governance, previous experience developing and implementing physical and cyber assessments applicable to a wide spectrum of critical infrastructure sectors provides a validated point of departure for a cyberbiosecurity assessment framework. This approach proposes to integrate existing capabilities and proven methodologies from the infrastructure assessment realm (e.g., decision science, physical security, infrastructure resilience, cybersecurity) with new expertise and requirements in the cyberbiosecurity space (e.g., biotechnology, biomanufacturing, genomics) in order to forge a flexible and defensible approach to identifying and mitigating vulnerabilities. Determining where vulnerabilities reside within cyberbiosecurity business processes can help public and private sector partners create an assessment framework to identify mitigation options for consideration that are both economically and practically viable and ultimately, allow them to manage risk more effectively.

## Introduction

An important initial step in effectively managing risk is developing a comprehensive understanding of vulnerabilities. Stakeholders can then identify economical and practical options to mitigate vulnerabilities. Risk in the biological sciences has been managed through the implementation of standard biosecurity practices, through which vulnerabilities are (a) identified and (b) mitigated through regularly updated training, policies, and enhanced physical security. To prevent unauthorized access to high-consequence biological agents, the U.S. Government (USG) stood up the Federal Select Agent Program (FSAP), which added extensive requirements (e.g., background checks, registration by institutions, increased oversight) for those seeking access to Biological Select Agents and Toxins (BSATs). The BSAT list is based on taxonomic classifications and includes 67 high-consequence biological agents and toxins. Advances in genetic engineering tools (e.g., CRISPR Cas 9 systems) along with the convergence of lab automation, computational biology, and access to publically available genomic databases will dramatically impact the effectiveness of the FSAP as well as other biosecurity policies and practices. It will no longer be necessary to obtain physical samples to exploit a biological agent; access to publically available genomic databases, biofoundries, lab automation, and computational biology enables the design and production of high-consequence biological agents and toxins. These biological agents may be entirely new to nature and unconstrained by taxonomic classification such as the BSAT list (Wintle et al., 2017). This new digital environment in which biological research increasingly takes place must be systematically assessed for vulnerabilities in order to effectively manage evolving risks. The new discipline of cyberbiosecurity, which includes biosecurity, cyber-physical security, and cybersecurity, directly addresses the unique risks associated with biotechnology in an increasingly digital environment (Peccoud et al., 2017; Murch et al., 2018).

In this paper, we outline the foundation of an assessment framework for cyberbiosecurity, accounting for both security and resilience factors in the physical and cyber domains. When implemented, the assessment framework will help partners identify and prioritize vulnerabilities. Importantly, the prioritization of vulnerabilities will result from a defensible, transparent, and reproducible assessment. In conjunction with an understanding of the consequences of disruption, risk mitigation strategies can be developed and considered in return-on-investment (ROI) analyses. ROIs will allow stakeholders to make informed decisions on how best to allocate limited resources for maximum impact.

While biosecurity is one of the three disciplines comprising cyberbiosecurity (e.g., biosecurity, cyber-physical security, and cybersecurity) it is well-established and will not be discussed due to space limitations.

## Risk Mitigation in the Era of Converging Technologies

Emerging and converging technologies present new risks to security that require new methodologies for risk prioritization and mitigation.

The accelerated pace of technological advancements across nearly all scientific disciplines has been driven largely by the convergence of advancements in scientific disciplines associated with computation, networking, automation, and access to data. Convergence occurs where scientific disciplines or key enabling technologies combine with other disciplines or enabling technologies and promise new or improved capabilities. Convergence is more than the simple combination of different disciplines or technologies. It leads to synergies, adding more value through convergence (Dengg, 2018).

While converging technologies lead to fast and far-reaching improvements, they also create new security challenges and risks. We often try to address new risks with methods that were successful in the past; however, they may not be appropriate for the systemic risks posed by the increasing interconnectivity and complexity associated with converging technologies (Dengg, 2018). Additionally, with highly interconnected systems, the risk from dependencies and interdependencies must be considered. Therefore, we must take a more systemic approach to assessing and mitigating risks resulting from converging technologies.

Emerging and converging technologies have significantly increased the number of vulnerabilities to national security to levels that are untenable for the government and private sector to address in their entirety. They simply do not have the resources required to implement mitigation strategies to address risks with a low probability of occurrence and/or low consequence. Current conversations do not prioritize potential courses of action based on defensible integrated risk assessments that consider both probability and consequence in the context of converging technologies.

## Cyberbiosecurity

The exploration of life sciences has become increasingly dependent upon internet-connected machinery and devices. Internet-dependent infrastructure is critical to computation and discovery of new avenues of research. The subsequent dependence upon technology and internet-connected devices begs the need to secure this infrastructure. For example, attackers could exploit unsecured networks and remotely manipulate biological material, creating new threats with devastating potential (Murch et al., 2018). Cyberbiosecurity aims to understand and reduce the risks associated with conducting research using advanced technologies in the bioscience field. Science exploration depends increasingly upon cloud services, cyber-physical devices, internet-connected machines, remote databases, and many other cyber-vulnerable technologies. This convergence of science and cybersecurity opens the field to a new threat landscape.

Below are two examples of vulnerabilities that may not be individually identifiable in either a biosecurity or a cybersecurity context but are only apparent when both disciplines are considered.

Bringing together advances in synthetic biology and genetic engineering with machine learning, advanced modeling, metabolic engineering and access to publically available databases containing complete genome sequences of pathogens including virulence factors will enable the design of novel high consequence biological agents completely *in silico*. Minimal laboratory infrastructure and equipment would be required. Moreover, the vast array of publically available open source tools enable execution of these processes by less experienced personnel.

Advances in laboratory automation have enabled tacit knowledge (e.g., hands-on know-how), traditionally requiring years of professional laboratory training, to be codified into executable code controlling automated laboratory equipment. The ability of automated laboratory equipment to reproducibly perform tasks once limited to well-trained laboratorians has been monetized in the form of commercial biological production facilities (e.g., biofoundries). These biofoundries may unwittingly produce components of high consequence biological agents solely from digital information provided by the customer. To request synthesis services, the customer simply goes to the website of the biofoundry and uploads the required biological data (e.g., DNA sequences, amino acid sequences, etc.). To obscure the identity and/or functional properties of the final product several biofoundries can be used, each synthesizing seemingly innocuous products representing only a portion of the final product.

Furthermore, contributions to the exploration of science are built upon the open and sharing nature of samples and knowledge. This inherent openness and trust that exist in the scientific community is ripe for exploitation (Peccoud et al., 2017). In order to thwart attackers and keep data secure, it is paramount that the Confidentiality, Integrity, and Availability (CIA triad) of scientific data is upheld in this digital era. Compromising any of the pillars within the CIA triad could lead to unwanted consequences. For example, attackers could:

- Exploit vulnerable infrastructure and steal proprietary sequences from a biotechnology firm, ruining the *confidentiality* of the stolen intellectual property;
- Manipulate DNA sequences for malicious intent, thereby destroying the *integrity* of a given sample or changing a sample to be something other then what is intended; or
- Degrade systems, compromising the *availability* of cyber-physical devices that are used to perform needed functions.

Ensuring the confidentiality, integrity, and availability of both the physical material and the associated digital information is essential to ensuring the safety and security of scientific advances in bioscience.

## Understanding Key Terms

Defining the key elements of the emerging field of cyberbiosecurity is important to ensuring a common understanding of the relevant technical issues that arise from this new hybrid discipline. It is equally important to define key terms related to risk, particularly for audiences that may not already be familiar with the core concepts relevant to biosecurity; cyber-physical security; and cybersecurity assessments, policies, and practices. An important foundational document in this regard is the *DHS Risk Lexicon*, published in 2010 by the U.S. Department of Homeland Security to level-set terminology across the homeland security enterprise (U.S. Department of Homeland Security (DHS), 2010).

As framed in the *DHS Risk Lexicon*, risk is the potential for an unwanted outcome resulting from an incident, event, or occurrence, as determined by its likelihood and the associated consequences. Evaluating the probability of adversarial attacks is challenging due in part to the lack of historical data in which to ground quantitative estimates, inability to project that future deliberate threats will resemble those of the past and the inherent challenges in evaluating the intent and capability of entities seeking to exploit weaknesses. Thus, risk in the Homeland Security space has been framed as a function of three elements: the threats to which an asset or system is susceptible; the vulnerabilities of the asset or system to the threat; and the potential consequences arising from the degradation of the asset or system. Each of these elements is defined below (U.S. Department of Homeland Security (DHS), 2010).

- Threat: natural or human-caused occurrence, individual, entity, or action that has or indicates the potential to harm life, information, operations, the environment, and/or property.
- Vulnerability: physical feature or operational attribute that renders an entity open to exploitation or susceptible to a given hazard.
- Consequence: the effect of an event, incident, or occurrence. Consequence is commonly deconstructed and measured in four categories: human, economic, mission, and psychological.

When talking about risk, it is also important to define what a hazard is due to its direct correlation and impact on vulnerabilities, threats, and consequences of an asset. A hazard is a natural or man-made source or cause of harm or difficulty. Threats are typically directed at an entity, asset, system, network, or geographic area, while a hazard is a natural or accidental phenomenon that is not driven consciously by an adversary.

Although not typically identified as one of the three core factors driving risk, resilience is an additional consideration that impacts assessments of risk and ensuing strategies for managing it. As a result, it is relevant to understanding ways to evaluate cyberbiosecurity. Resilience is the ability to resist, absorb, recover from or successfully adapt to adversity or a change in conditions (U.S. Department of Homeland Security (DHS), 2010). Resilience features play a role in both the vulnerability and consequence variables in risk. Resilience measures can reduce vulnerability to various threats and hazards through protective measures that improve an organization's ability to resist an event or absorb its effects with minimal impact. Similarly, on the consequence side, resilience measures can enhance an entity's ability to quickly adapt and respond to an incident, as well as to recover and return to normal operations more quickly (U.S. Department of Homeland Security (DHS), 2010; Petit et al., 2013b).

**Figure 1:**
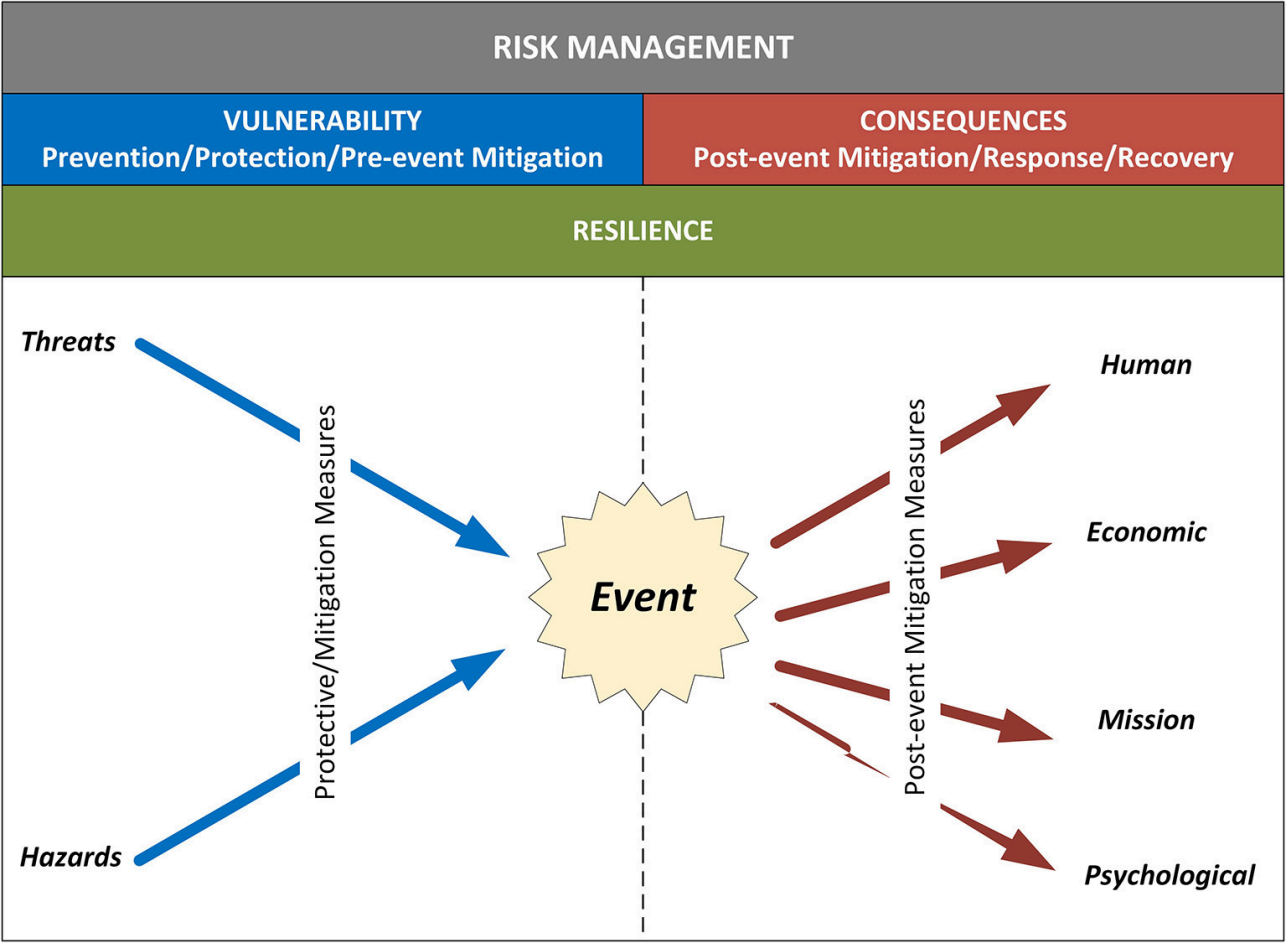
Risk management. By understanding the likelihood of various threats and hazards, associated vulnerabilities, potential consequences, and resilience characteristics, stakeholders can make informed decisions on ways to manage risk (i.e., accept, transfer, avoid, or mitigate).

Taking into consideration all of these inputs, organizations can institute defensible, repeatable, and actionable processes to analyze risk and ultimately, to make decisions on how to manage it. Risk management is the process of identifying, analyzing, and communicating risk and then accepting, avoiding, transferring or controlling it to an acceptable level and at an acceptable cost (U.S. Department of Homeland Security (DHS), 2010). Risk management involves knowing the threats and hazards that could potentially impact a given organization, the vulnerabilities that render it susceptible to particular hazards, and the various consequences that might result. Figure 1 illustrates how these various components combine to drive risk-based decision-making (Petit et al., 2013a).

Cyberbiosecurity is a new field that brings together different disciplines in new ways, triggering a pressing need for new thinking in terms of relevant threats, vulnerabilities, and consequences. Existing approaches used in biosecurity, cyber-physical security, and cybersecurity communities provide important foundational concepts and organizing principles, but they do not adequately capture emergent features related to biological and biomedical systems. Biosecurity, cyber-physical security, and cybersecurity are defined below.

- Biosecurity: describes the protection, control and accountability of biological materials in order to prevent their unauthorized access, loss, theft, misuse, diversion or intentional release.
- Cyber-physical security: addresses the potentially high-consequence dependency between physical systems and the special-purpose computers that control and monitor them.
- Cybersecurity: addresses the risks of computer and network systems used for managing processes and sharing and protecting information.

## Considering Dependencies and Interdependencies in Cyberbiosecurity

In addition to the concepts defined in the previous section, another concept that is relevant to understanding risk—including but not limited to the cyberbiosecurity domain—is the notion of how dependencies and interdependencies among and between complex systems impact overall risk. Dependencies and interdependencies are key to how the public and private sector understand, analyze, and manage risk within and across critical infrastructure sectors and other complex systems.

A dependency is a *unidirectional relationship* between two assets, in which the operations of one asset affect the operations of the other. For example, a water treatment plant may depend on an external data source to process its water for potability. An interdependency is a *bidirectional relationship* between two assets, in which the operations of both assets affect each other. For example, the water treatment plant requires communications for its supervisory control and data acquisition (SCADA) system, and, in turn, provides water used by the communications system to cool its equipment. An interdependency is effectively a combination of two dependencies—therefore, understanding an interdependency requires analyses of the one-way dependencies that comprise it (Petit et al., 2015).

Effective analysis of dependencies and interdependencies (whether for critical infrastructure, cyberbiosecurity, or other fields of study) requires some basic frameworks for defining, categorizing, and characterizing key features. For example, since infrastructure systems are constantly interacting with their environment and using inputs to generate outputs, it is important to identify where a dependency or interdependency exists within this activity chain. Upstream dependencies are the products or services provided to one system by an external source that are necessary to support its operations and functions. Internal dependencies involve interactions among internal operations, functions, and missions of the system. Downstream dependencies speak to the consumers or recipients who rely on the system's output and are affected by service disruptions or resource degradation (Petit et al., 2015).

Dependencies and independencies are effectively risk multipliers—they can amplify vulnerabilities and consequences that arise from different threats and hazards. For example, loss of a service such as electric power can potentially affect other infrastructure systems that require power to operate, exacerbating the effects of the original power outage and possibly triggering other unanticipated downstream impacts. The presence of dependencies and interdependencies within the cyberbiosecurity domain make the already complex task of understanding risk that much more complicated, requiring analysts not only to evaluate threat, vulnerability, and consequence factors, but also to characterize relevant dependencies and interdependencies that can render complex systems more susceptible to disruption or exploitation.

## Focusing on Vulnerability

While the field of cyberbiosecurity is new, community members can leverage extensive knowledge and applications from other fields in order to begin stitching together an overarching framework for understanding relevant threats, vulnerabilities, and consequences from a cyberbiosecurity perspective, whether at a facility, system, or organizational level.

Biolabs need an assessment toolkit that: (1) apply to a wide range of assets and systems across different sectors; (2) produce repeatable, defensible, and actionable results; (3) balance the need for efficiency with the need for detailed data; and (4) build on sound scientific principles, industry standards, and recognized best practices. The approaches above have been used to build and deliver multiple infrastructure assessment tools focused on vulnerability (e.g., Infrastructure Survey Tool (IST), Cyber Infrastructure Survey Tool (Cyber IST), Modified Infrastructure Survey Tool) and are based on the principles of decision analysis, an approach that can be used to manage risk under conditions of uncertainty (Keeney and Raiffa, 1976; Kenney, 1992). When combined with additional analyses that evaluate potential threats and consequences of disruptions or loss, these processes can help biosecurity partners understand their broader risk environment and potential courses of action to mitigate risk.

One example application that could be helpful to the biosecurity community is the IST, which DHS field personnel use to evaluate security and resilience at critical infrastructure facilities nationwide in partnership with infrastructure owners and operators. The IST includes an index—the Protective Measures Index (PMI)—that characterizes the protective measures posture of individual facilities based on their most vulnerable aspects (Fisher et al., 2009; Petit et al., 2011). The PMI aggregates data collected through a structured onsite assessment process into four levels of information (or subcomponents) across five major categories. For each subcomponent, an index corresponding to the weighted sum of its subcomponents is calculated. This process results in an overall PMI that ranges from 0 (low protection) to 100 (high protection) for the critical infrastructure analyzed, as well as index values for various subcomponents (Petit et al., 2013b).

The decision analysis methodology used to define the PMI was specifically developed to integrate the major elements that are relevant to protecting critical infrastructure. The methodology integrates physical elements that are traditionally part of protection analysis (e.g., fencing, gates, entry controls, intrusion detection systems) as well as operational elements (e.g., security management, security planning, information-sharing mechanisms). The process for identifying specific security characteristics that contribute to protection at a facility and then establishing relative weights required a series of structured elicitation sessions with subject matter experts from public and private sectors (Petit et al., 2013a).

Ultimately, organizing PMI components into different levels and ranking their relative importance allows for the creation of reproducible results and visually compelling outputs that help owners and operators of critical infrastructure make tradeoff decisions on potential courses of action. Furthermore, the use of a consistent index and the consistent deployment of the toolset for a decade has allowed users to compare their results with other assets in the same sector.

Another example that could be helpful to analysis is the Cyber IST, which focuses on critical cyber services. A cyber service is any combination of equipment and devices (hardware), applications and platforms (software), communications, and data that have been integrated to provide specific business services. In this case that would classify as lab systems whose loss would result in physical destruction, safety, and health effects (e.g., a chemical release or loss of environment controls); theft of sensitive information that can be exploited; business interruption (e.g., denial of service); or other economic loss to the organization or its customers/users. The Cyber IST generates a Cyber Protection and Resilience Index (CPRI) as its mechanism for organizations to use in comparative analysis.

In cybersecurity, identified threats, vulnerabilities, and consequences are often categorized into how these risks affect the confidentiality, integrity, and availability of a critical cyber service. These factors are considered the three most significant elements of reliable cybersecurity. Confidentiality limits who has access to information. Integrity governs how and when information is modified. Availability is the assurance that people who are authorized to access the information are able to do so. The question set for the Cyber IST was developed by subject matter experts based on the CIA triad, to assess how businesses help uphold the Confidentiality, Integrity, and Availability of their critical cyber services (Joyce et al., 2017). This same question set provides the basis for assessing confidentiality, integrity, and availability of critical cyber services or assets within the context of cyberbiosecurity.

## Considering the Human Factor in Cyberbiosecurity

Insiders pose substantial threats to cyberbiosecurity because they already have authorized access to critical systems. Most security measures are designed to protect the organization from external attacks and are often more difficult to implement to protect from internal attacks. The potential consequences of threats from insiders vary by the amount of trust and authority given to them (Evans, 2009).

Insiders include not only employees of the organization but also employees of trusted business partners, if those partners have access to the organization's systems, equipment, or data. The threats posed by insiders include both unintentional and intentional, both of which should be accounted for in cyberbiosecurity assessment frameworks. Unintentional incidents often result from negligence or misjudgment. Intentional incidents include insiders who commit fraud for financial gain or seek to sabotage the organization.

Both unintentional and intentional insider incidents can result from actions taken by external actors. For example, unintentional insider incidents may involve insider personnel responding to phishing or social engineering attacks from outside parties, while intentional incidents could involve personnel colluding with external actors, either voluntarily or under pressure. Insiders could willingly participate based on involvement in a cause or support to foreign government or organization, or they may fall victim to recruitment by a criminal enterprise either because of financial or personal troubles (Perkins and Fabregas, 2018).

## Roadmap for a Cyberbiosecurity Assessment Framework

Moving forward, the diverse community of researchers and practitioners in the cyberbiosecurity domain should collaborate to establish a common vulnerability assessment framework that is grounded in decision science; apply lessons learned from parallel efforts in related fields; and reflect the complex multidisciplinary cyberbiosecurity environment. Key steps in this process should include:

- Engaging subject matter experts in decision science, biotechnology, biosecurity, cyber-physical security, cybersecurity, and physical security in a collaborative assessment development process.
- Defining functional requirements of assessment processes to ensure common understanding of goals, objectives, and constraints.
- Characterizing the biotechnology ecosystem based on facility type (e.g., universities, biofoundries, pharmaceutical companies) and supporting systems (e.g., bioprocess, supply chain, supporting information systems, facility infrastructure) to identify likely assessment candidates and pathways.
- Identifying relevant industry standards, legal frameworks, and regulatory regimes that apply to cyberbiosecurity.
- Establishing a comprehensive taxonomy of characteristics in physical assets and cyber systems in the biotechnology community that influence security posture (e.g., access control, security management, personnel, response protocols, dependencies).
- Conducting an iterative elicitation process to establish subject matter expert consensus on relative importance of security characteristics and their subcomponents in order to facilitate data aggregation, comparison with like entities, and alternatives analysis.
- Exploring potential approaches for collecting assessment data and visualizing assessment results.

## Author Contributions

DS conceived the manuscript and all authors have jointly contributed to the manuscript, with particular contribution of L-AL to the text on the cyberbiosecurity assessment framework and dependencies. Contribution of NE to the text on cybersecurity and the human factor. All authors have read and approved the manuscript for publication.

### Conflict of Interest Statement

The authors declare that the research was conducted in the absence of any commercial or financial relationships that could be construed as a potential conflict of interest.
